# Modelling the Characteristics of Ring-Shaped Magnetoelastic Force Sensor in Mohri’s Configuration

**DOI:** 10.3390/s20010266

**Published:** 2020-01-02

**Authors:** Anna Ostaszewska-Liżewska, Roman Szewczyk, Peter Raback, Mika Malinen

**Affiliations:** 1 Warsaw University of Technology, Faculty of Mechatronics, Institute of Metrology and Biomedical Engineering, sw. A. Boboli 8, 02-525 Warsaw, Poland; a.ostaszewska@mchtr.pw.edu.pl; 2 CSC–IT Center for Science, P.O. Box 405, FI-02101 Espoo, Finland; Peter.Raback@csc.fi (P.R.); Mika.Malinen@csc.fi (M.M.)

**Keywords:** magnetoelastic effect, force sensor, finite element method

## Abstract

Magnetoelastic force sensors exhibit high sensitivity and robustness. One commonly used configuration of force sensor with a ring-shaped core was presented by Mohri at al. In this configuration force is applied in the direction of a diameter of the core. However, due to inhomogeneous distribution of stresses, model of such sensor has not been presented yet. This paper is filling the gap presenting a new method of modelling the magnetoelastic effect, which is especially suitable for the finite element method. The presented implementation of proposed model is in good agreement with experimental data and creates new possibilities of modelling other devices utilizing magnetoelastic effect.

## 1. Introduction

Magnetoelastic stress and force sensors are promising solution for measurements in mechanical systems operating in hard industrial conditions. Such sensors utilize the dependence of magnetic characteristics of soft magnetic materials on the mechanical stress *σ* [1,2], especially changes of relative permeability *μ_r_*(*σ*) of material. One of emerging direct *μ_r_*(*σ*) approaches is the SI (Stress-impedance) effect [3] and change of magnetic anisotropy direction [4], while advanced material studies show magnetomechanical influence on unbalanced small angle magnetization rotation (SAMR) [5], Matteucci [6], or even thermoelectric [7] voltage. Due to their robustness, sensitivity and reliability [8,9] magnetoelastic sensors are intensively developed for the most demanding industrial [10] and biomedical [11] applications.

**Figure 1:**
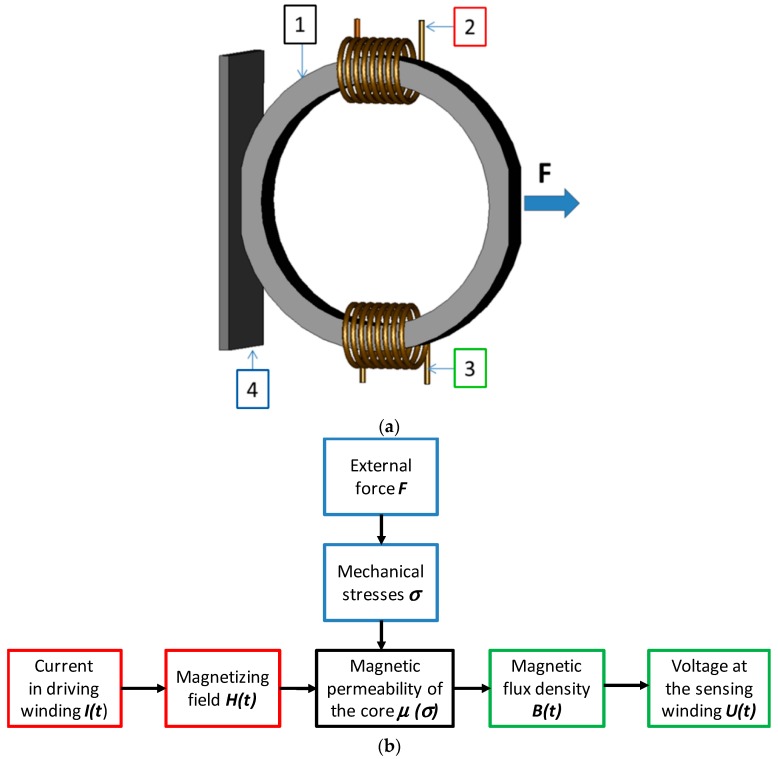
Magnetoelastic sensor in Mohri’s configuration: (**a**) general view of a ring-shaped sensor [8]: 1–a magnetoelastic core, 2–a driving winding, 3–a sensing winding, 4–a base plane; (**b**) schematic diagram explaining the principles of operation of the sensor.

A magnetoelastic sensor in a configuration presented by Mohri et al. utilizes a ring-shaped core with compressive and tensile forces applied in the direction of its diameter [12,13]. Such a magnetoelastic sensor is one of the most common solutions used in the industrial practice. The schematic view of Mohri’s magnetoelastic sensor is presented in Figure 1a whereas schematic diagram explaining the principles of operation of such sensor is presented in Figure 1b.

It should be highlighted that mechanical stress distribution in the core of the magnetoelastic sensor in Mohri’s configuration is inhomogeneous. Moreover, both compressive and tensile stresses occur in the core. As a result, the quantitative model of such a sensor was not still presented.

This paper is filling the mentioned gap in the state of the art. New idea of modelling the stress dependence of the relative permeability tensor *μ_r_* is proposed. On the base of this idea, the finite element model of the magnetoelastic sensor in Mohri’s configuration is presented. The model is implemented in an open-source Elmer environment, enabling the verification of experimental characteristics and further optimisation of magnetoelastic sensors.

## 2. Proposed Model of Stress Dependence of Magnetic Relative Permeability Tensor for 2D Stress Distribution

The magnetoelastic effect is connected with the changes of total free energy of a magnetic material subjected to mechanical stresses [14]. Magnetoelastic energy *E_σ_* for isotropic magnetic materials (e.g., amorphous or polycrystalline materials where stress and elasticity is approximately isotropic) is given by the following Equation (1) [15]:

(1) $$E_{\sigma }=-\frac{3}{2}\lambda \sigma \left(sin^{2}\beta -\upsilon \cdot cos^{2}\beta \right)$$

where *ν* is the Poisson ratio, *λ* is magnetostriction, *σ* is the mechanical stress and *β* is the angle between *λ* and *σ*. As a result it can be observed that for materials with positive saturation magnetostriction *λ_s_* subjected to tensile stresses *σ*, the value of relative permeability *μ_r_* is increasing, whereas for compressive stresses it decreases. The increase of relative permeability *μ_r_* is limited by Villari reversal [16], where saturation magnetostriction changes its sign [17]. However, the quantitative relation between magnetoelastic energy *E_σ_* and relative permeability *μ_r_* in the material is not obvious. In general it can be explained on the base of anisotropic extension of anhysteretic model of magnetization in Jiles-Atherton model. In such a case, magnetoelastic energy *E_σ_* should be treated as anisotropic energy *K_an_* as it was presented previously [18].

Moreover, as it was proven previously [19,20], the influence of a perpendicular stress $\sigma_{⊥}$ (generated by external forces) on the magnetoelastic characteristic is given in terms of *σ_e_* defined by the following Equation (2):

(2) $$\sigma_{e}=-\nu \cdot \sigma_{⊥}$$

where *ν* is the Poisson ratio.

On the other hand, for limited values of mechanical stresses *σ*, for amorphous alloy Fe_25_Ni_55_Si_10_B_10_, considering the Figure 4 in [21] (for the assumption that magnetizing field was H = 50 A/m) the mechanical stress *σ* dependence of relative permeability *μ_r_* can be estimated by a linear Equation (3):

(3) $$\mu_{r}\left(\sigma \right)=14540-573\;\cdot \sigma \;\left(\mathrm{MPa}\right)$$

Please note that in the Equation (3), uniaxial stresses *σ* are expressed in MPa. Moreover, for soft magnetic materials, linear approximation of *μ_r_*(*σ*) relation may be used only for limited value of stresses, whereas for higher values of stresses Villari maximum may occur. In such a case linear relation is not suitable to describe the stress dependence of magnetic permeability. Spline empirical interpolation is recommended instead.

In inhomogeneous stress distributions, both axial and shear stresses may occur. However, in such a case, principal stresses *σ_P_* = [*σ_Px_ σ_Py_ σ_Pz_*] may be calculated. Principal stresses *σ_P_* are the only components of the stress tensor, when the local coordinate system is changed, leading to a reduction of shear stress components to zero [22]. Vanishing of shear stress is connected with principal stresses concept. This mechanical phenomena is not dependent on symmetry. Moreover, principal stresses can be calculated for all shear–axial stresses systems. As a result only information about permeability dependence on axial stresses is necessary to model any mechanical system.

**Figure 2:**
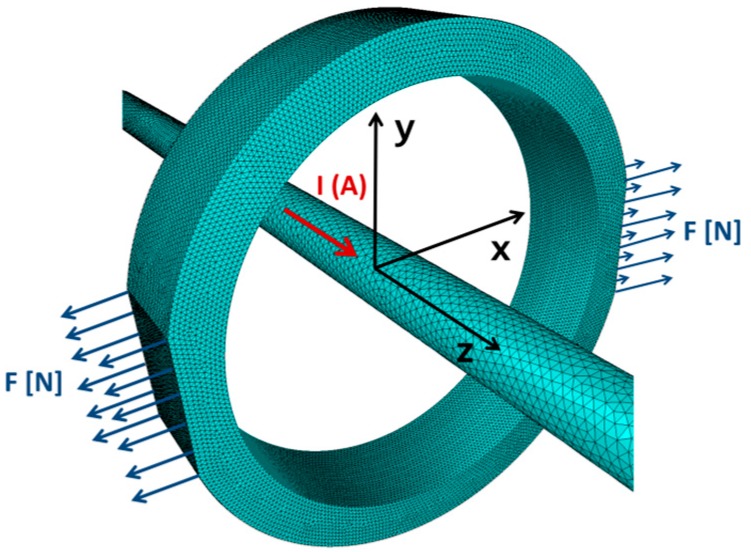
Simplified model of a magnetoelastic sensor in Mohri’s configuration. F–a measured force, I–a magnetizing current.

Figure 2 presents the simplified model of the magnetoelastic sensor in Mohri’s configuration, where the ring core is driven by the straight wire instead of magnetizing winding. The outside diameter of the sample was 30 mm, inside diameter was 25 mm, whereas height was 7 mm. The magnetoelastic core is magnetized by current *I* in an axial wire. It should be highlighted that in the presented model both mechanical stresses *σ* and a flux density *B* in the direction of Z axis can be neglected. As a result, the presented case can be reduced to a 2D system.

In the case presented in Figure 2, principal stresses *σ_P_* for the 2D stress distribution occur in X-Y plane, whereas the coordinate system is rotated by an angle *ϕ* around the Z-axis. As it was indicated above, such a rotation of the coordinate system leads to vanishing shear stresses [23].

As a result, the relative permeability tensor *μ_r_* determined by uniaxial anisotropy induced by principal stresses *σ_P_* in isotropic magnetic material equals to:

(4) $$\mu_{r}=R*\left[\begin{array}{ccc} \mu_{r}\left(\sigma_{Pxx}\right) & 0 & 0\\ 0 & \mu_{r}\left(\sigma_{Pyy}\right) & 0\\ 0 & 0 & \mu_{r}\left(\sigma_{Pzz}\right) \end{array}\right]*R^{-1}\;$$

where *R* is the rotation matrix for the angle *ϕ* around the Z-axis:

(5) $$R=\left[\begin{array}{ccc} cos\;\phi & -sin\;\phi & 0\\ sin\;\phi & cos\;\phi & 0\\ 0 & 0 & 1 \end{array}\right]$$

Considering the fact, that stresses in the Z direction are negligible, efficient stresses *σ_Pxx_* and *σ_Pyy_* can be calculated form the principal stresses *σ_Px_* and *σ_Py_*, according to the Equation (2):

(6) $$\sigma_{Pxx}=\sigma_{Px}-\nu \sigma_{Py}\;$$

(7) $$\sigma_{Pyy}=\sigma_{Py}-\nu \sigma_{Px}\;$$

Finally, the magnetic flux density *B* vector is calculated considering the relative permeability *μ_r_* tensor:

(8) $$\vec{B}=\mu_{0}\mu_{r}\times \vec{H}$$

where *μ*_0_ is the magnetic constant. It should be highlighted, that vector product of 3 × 3 *μ**_r_*** permeability tensor and $\vec{H}$ vector is $\vec{B}$ vector, accordingly to the matrix multiplication formalism.

## 3. Modelling the Magnetoelastic Sensor in ELMER FEM Environment

For the modelling presented in the paper only the open-source software was used. As a result a validation of the calculation algorithm was possible. To enable validation and further development of the model, sources required for modelling are available on MIT licence at www.github.com/romanszewczyk/FEM in the “Mohri” subdirectory. Moreover, proposed models can be used for commercial applications without additional costs of licences.

Tetrahedral meshes for modelling were generated with NETGEN 6.2 software [24] utilizing Delaunay method. The maximal height of tetrahedral elements of the ring-shaped core was 0.7 mm, whereas the maximal height of tetrahedral elements of the wire was 0.5 mm. It should be highlighted, that NETGEN 6.2 enables creation of meshes from the text style. geo file in the batch mode, what enables automatic changes of mesh geometry by other software.

The whole modelling was controlled by OCTAVE 5.0 software, which is efficient MATLAB alternative. OCTAVE generated the. sif file, which determines the finite elements model for ELMER FEM software [25]. For the modelling five solvers were used: *StressSolver* for mechanical modelling, *StatCurrentSolver* to determine the current flow in the driving wire and *WhitneyAVSolver* together with *MagnetoDynamicsCalcFields* for magnetic modelling. Finally, the results of simulation, both magnetic flux density *B* vector distribution as well as mechanical stresses *σ* tensor were exported to text file using *SaveGridData* solver. It should be highlighted, that Equations (3)–(7) were implemented in the model using *MATC* language built into the Elmer FEM software.

The analysis of the modelling results was carried out with OCTAVE 5.0 software on the base of output text file, generated by the *SaveGridData* solver of ELMER FEM. Visualization of the results was done using QT graphics toolkit implemented in OCTAVE 5.0.

## 4. Results of Modelling in Comparison with Experimental Results

**Figure 3:**
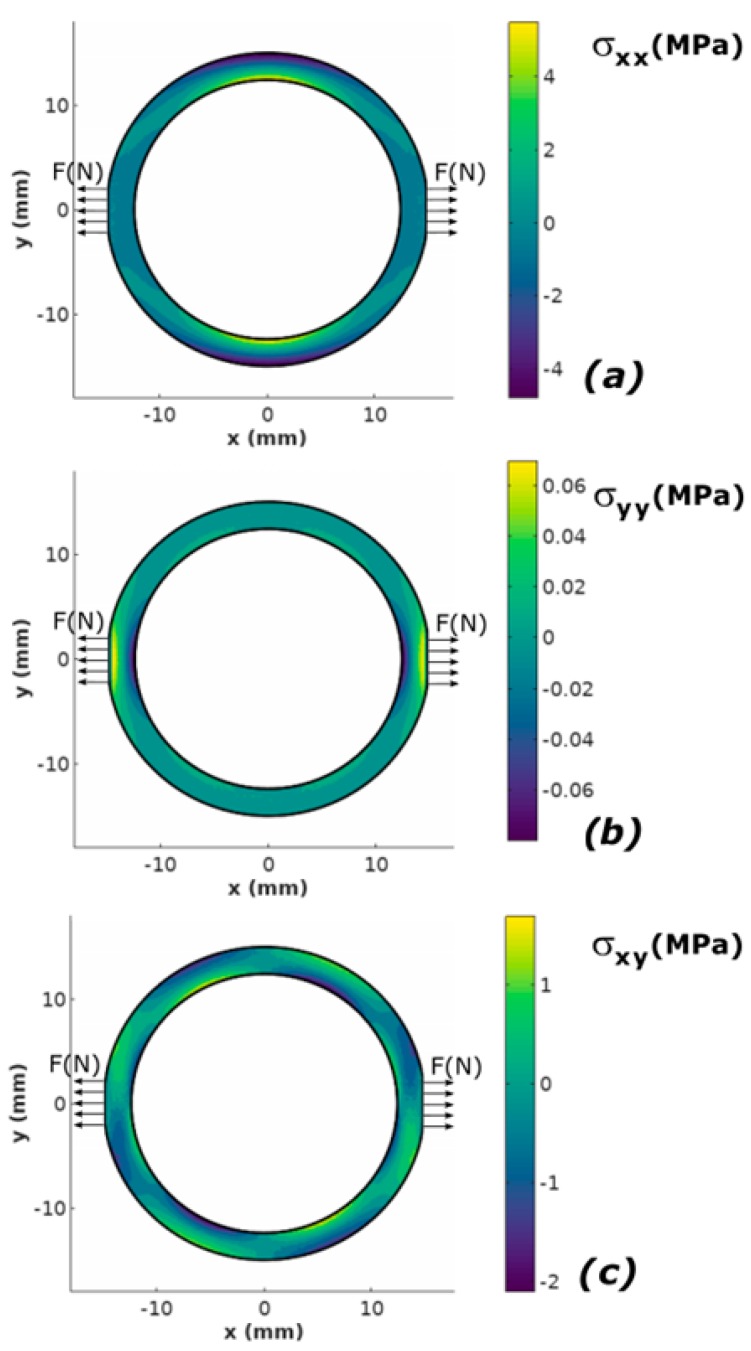
The mechanical stress distribution in the ring-shaped core of the magnetoelastic sensor in Mohri’s configuration, loaded by the measured tensile force F: (**a**) an axial stress *σ_xx_*, (**b**) an axial stress *σ_yy_*, (**c**) the shear stress *σ_xy._*

Mechanical axial stresses *σ_xx_* and *σ_yy_*, as well as shear stresses *σ_xy_* distribution in the ring-shaped core of the magnetoelastic sensor in Mohri’s configuration, loaded by the measured tensile force *F*, is presented in Figure 3. As expected it can be observed that both tensile and compressive stresses occur in the core of the magnetoelastic sensor.

**Figure 4:**
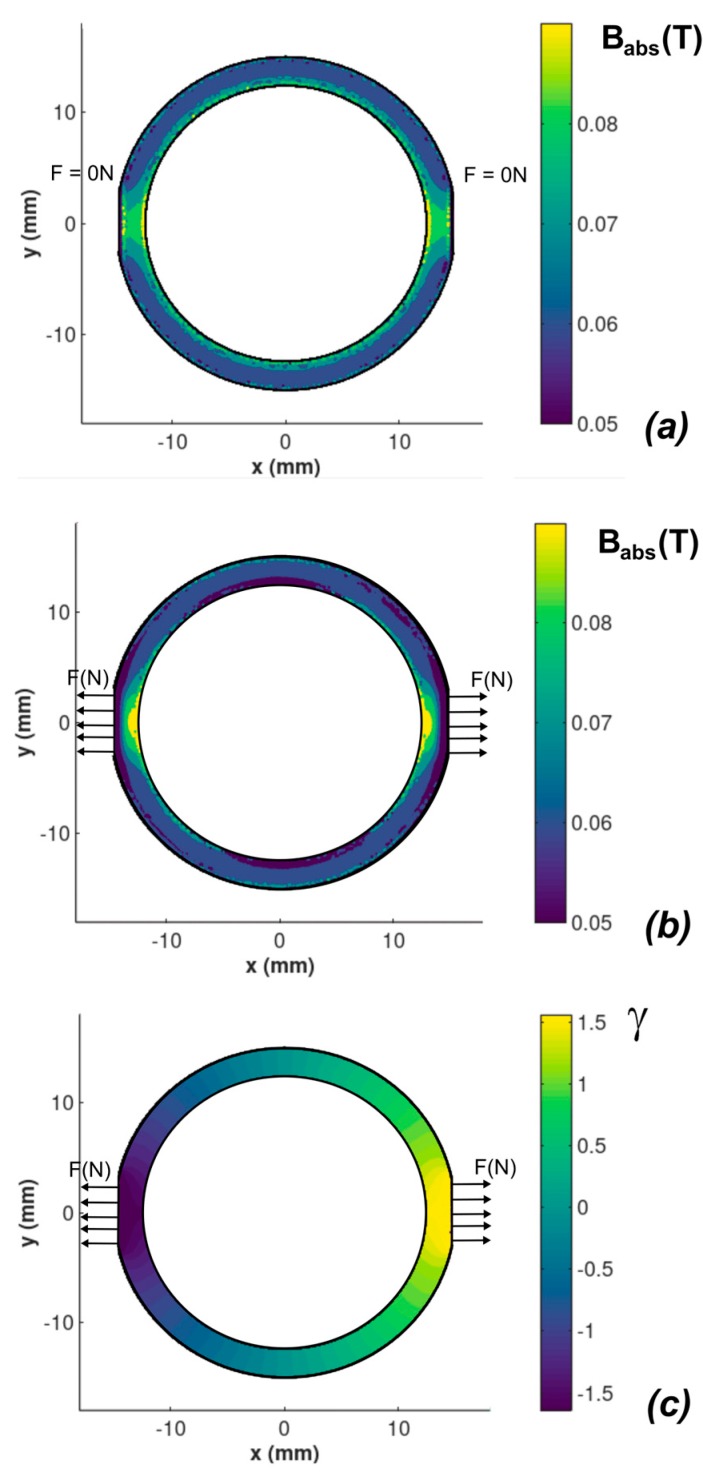
The flux density *B* distribution in the core of the magnetoelastic sensor subjected to mechanical stresses generated by the force *F*, (**a**) absolute length of the flux density *B* vector for sample not subjected to stresses (*F* = 0 N), (**b**) absolute length of the flux density *B* vector for sample subjected to stresses *F* = 50 N, (**c**) in *γ* between the flux density *B* and x-axis for sample subjected to stresses *F* = 50 N.

Figure 4 presents the flux density *B* distribution in the core of magnetoelastic sensor. Without external forces, the flux density *B* distribution would be symmetric and uniform as it is presented in Figure 4a. It can be observed that the influence of mechanical stresses significantly influences on the flux density in the core (Figure 4b). However, due to the inhomogeneous mechanical stress distribution, it is difficult to describe the dependence of stress on the distribution of the flux density *B*. As a result, for different sizes of magnetoelastic cores, modelling should be carried out for each case.

On the base of presented results of stress dependence of the flux density *B* in the core of the magnetoelastic sensor, the value of the amplitude of output voltage *U_out_* was calculated on a sensing winding (presented in Figure 1). For this calculation the numerical integration of flux density *B* in the ring-shaped core’s cross-section was used. Output voltage *U_out_* was calculated on the base of Faraday’s law:

(9) $$U_{out}\left(t\right)=n\cdot S\cdot \frac{dB\left(t\right)}{dt}$$

where *n* is the number of turns of sensing winding, whereas *S* is the field of core’s cross-section calculated from its dimensions.

**Figure 5:**
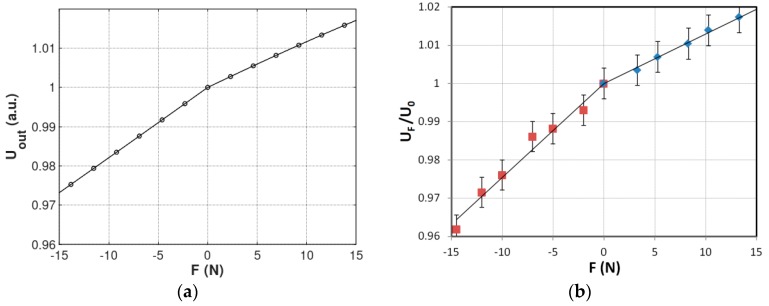
(**a**) The output voltage *U_out_* dependence on applied force *F* for the modeled magnetoelastic sensor in Mohri’s configuration, (**b**) the experimental results of the output voltage *U_F_/U*_0_ dependence on applied force *F.* (red points - compressive force, blue points - tensile force).

The results of modelling are presented in Figure 5a. The output voltage is given in arbitrary units due to the fact that it is dependent on the number of sensing winding turns as well as on geometry of the sensor. The explanation of the phenomenon of the different slopes of the characteristic presented in f for tensile and compressive force *F* is connected with the axial symmetry in magnetic flux density *B* presented in Figure 4a,b. Value of flux density *B* near the inner diameter of the core is higher, what interfere with inhomogeneous distribution of the stresses generated by the force *F*.

It should be highlighted that the results of modelling confirmed the experimental results presented by Mohri et al. Presented characteristic of the sensor is linear for both tensile and compressive forces. However, the results of modelling indicate different sensitivity of the sensor for compressive and tensile forces *F*. The difference between slopes of the curve is caused by the fact that the modelled sensor was magnetized by a single axial wire instead of a magnetizing winding. As a result asymmetry in stress distribution occurs for tensile and compressive stresses, what can be observed in Figure 3c.

In order to validate the modelling results, sample ring-shape core made of Fe_25_Ni_55_Si_10_B_10_ amorphous ribbon was tested in Mohri’s configuration. The core was in as-cast state, stabilized with cyanoacrylate adhesive in order to obtain rigidness, and had the same dimensions as modelled one. The sensing winding consisted of 100 turns, magnetizing winding was single axial wire. Sinusoidal magnetizing current of 50 Hz frequency from bipolar power supply (RMD-2b, WUT, Warsaw, Poland) was set to 50 A/m magnetizing field, and the output voltage was measured by the AC RMS voltmeter (Tonghui 1961, China). The compressive and tensile force was set with help of laboratory weights. For given magnetizing field, the voltage output for zero force was *U*_0_ = 144 mV. The results of the of the normalized output voltage *U_F_/U*_0_ dependence on applied force *F* is presented in Figure 5b.

## 5. Conclusions

Results presented in the paper confirm that the open-source software based on finite element method modelling can be efficiently used to describe the functional characteristics of magnetoelastic sensors. As a result, even sensors with an inhomogeneous distribution of mechanical stresses can be modelled with required accuracy.

Considering the fact that the axial and the shear stress system can be reduced to axial principal stresses in a rotated coordinate system, in the case if isotropic magnetic materials, even inhomogeneous systems can be modelled on the base of known dependence of relative permeability *μ_r_* on axial stresses *σ*. The presented linear approximation of *μ_r_*(*σ*) dependence can be developed in the future to consider also the Villari reversal as well as the magnetizing field dependence of relative permeability *μ_r_*.

In addition, it should be highlighted, that Mohri’s magnetoelastic sensor can also work with single wire excitation considering the changes of self-inductance. Such sensor configuration should be the subject of further research in the area of ring-shaped magnetoelastic sensors.
